# Access, autonomy, and affordability: ethical and human rights issues surrounding multigene panel testing for cancer in Japan and Switzerland

**DOI:** 10.3389/fgene.2024.1343720

**Published:** 2024-01-26

**Authors:** Kate Nakasato, Carlotta Manz, Kazuto Kato

**Affiliations:** ^1^ Department of Biomedical Ethics and Public Policy, Graduate School of Medicine, Osaka University, Osaka, Japan; ^2^ Centre of Comparative, European and International Law, University of Lausanne, Lausanne, Switzerland

**Keywords:** multigene panel testing, equal access, patient autonomy, healthcare affordability, genomic medicine, precision medicine, Japan, Switzerland

## Abstract

**Introduction:** Advancements in precision medicine and genomics have led to prospects in a wide range of clinical fields, including oncology. In particular, developments in next-generation sequencing multigene panel tests have led to the possibility of tailoring treatment to the specific genomic markers of a patient’s cancer. However, findings from current literature suggest that the path to implementation and uptake of genomic medicine is not without uncertainties and challenges.

**Methods:** To better understand the current challenges to the implementation of genomic medicine services, we investigated the current state of patient access to genomic medicine in Japan and Switzerland. In this investigation, we focused on equal access, patient autonomy, and healthcare affordability.

**Results:** Results have shown that although multigene panel testing is in principle covered by health insurance in both countries, barriers exist in terms of where the tests are available, comprehensive information for patients, and the affordability of not only the test itself but the overall process from diagnosis to treatment.

**Discussion:** These results suggest a need to continue examining a more diverse range of clinical landscapes for genomic medicine to reveal more nuanced understandings of barriers to implementation and thus better identify best practices for overcoming them.

## 1 Introduction

Before the emergence of precision medicine, medical practices were often based on evidence from the “average” patient. However, this “one-size-fits-all” approach does not work for everyone (National Institutes of Health NIH, 2023a). In contrast to this, precision medicine utilizes a patient’s genomic, environmental, and lifestyle information to more precisely prevent, diagnose, or treat disease (US Food and Drug Administration, 2018). The development of precision medicine has been accelerated by developments in the field of genomics, particularly the development of next-generation sequencing (NGS) tests that rapidly sequence large sections of an individual’s genome. Such tests include whole genome sequencing, whole exome sequencing, and multigene panel testing, which are being increasingly utilized in a wide range of clinical fields (Chakravarty et al., 2022).

In the field of oncology, cancers that have been traditionally classified and treated based on the area of the body in which they originated can now be divided into more specific sub-types based on their genomic makeup. Because not all cancers manifest and react to standard treatment in the same way, precision medicine allows physicians to identify the specific mutation that is causing a patient’s cancer and tailor their treatment accordingly, thus increasing chances of survival (Office fédéral de la santé publique OFSP, 2022a; National Institutes of Health NIH, 2023b). Identifying these mutations is commonly done through the use of multigene panel tests, which sequence a defined list of genes to identify the mutations that are causing the patient’s particular cancer. These tests are often referred to as companion diagnostics due to the supportive role they play in identifying the therapeutic drugs that may be used to best treat a patient’s cancer (Chakravarty et al., 2022).

Several countries have been pursuing national initiatives to promote the application of precision medicine into cancer care, i.e., cancer genomic medicine. Two well documented examples are the National Institutes of Health (NIH) All of Us Research Program from the in the United States and the National Health Services (NHS) Genomic Medicine Service from the United Kingdom. The All of Us program was established in 2015 with the aim of building a diverse database of genomic data to accelerate precision medicine, including cancer genomic medicine (National Institutes of Health NIH, 2020). The NHS Genomic Medicine Service from the United Kingdom was established in 2018 to become the first national health system to offer whole genome sequencing (WGS) as a part of routine care, including extended access to routine genomic testing to patients with cancer (National Health Service NHS, 2023). Other examples include Genomic Medicine France, which aims to sequence 235,000 genomes each year, primarily for caner and rare diseases” (Lévy, 2016), and Genomic Medicine Sweden, which was established in 2018 to promote the application of genomic technologies into clinical practice, including the use of whole genome sequencing for childhood cancer (Stenzinger et al., 2022; Genomic Medicine Sweden, 2023).

While such initiatives may hold potential, practical challenges have become apparent. In particular, current literature has highlighted numerous barriers to patient access of cancer genomic services. Two studies investigating the access to cancer genomic services in Europe have pointed out that availability and cost act as two key barriers, which applies not only to the testing itself but also the treatment that test results may indicate (Normanno et al., 2022; Bayle et al., 2023). In the US, where a significant proportion of the population lacks health insurance, studies have highlighted both system-level and patient-level barriers to patient access to cancer genomic services. These factors include cost and insurance coverage as well as patient literacy (Cooper et al., 2022).

Barriers such as the availability and cost of genomic services for cancer are influenced by a country’s healthcare system and other societal demographics. As such, in order to reveal more nuanced understandings of barriers to patient access and thus better identify best practices for overcoming them, there is a need to continue investigating a more diverse range of clinical landscapes. While several studies have been carried out in the contexts of the US and Europe, other regions are understudied. Furthermore, while comparative studies have often been carried out between countries that are similar in terms of regions, comparing countries with distinct situations from different parts of the world may identify shared challenges that are related to the implementation of cancer genomic medicine, regardless of a country’s healthcare or societal context. As an addition to the current state of knowledge, we chose to analyze and compare patient access to cancer genomic medicine, specifically multigene panel testing, in Japan and Switzerland.

At first glance, the differences between the two countries are clear. Japan is significantly larger than Switzerland, with a population of 126.5 million compared to Switzerland’s 8.5 million. Many institutions in Japan, including its healthcare, are centralized across the country (Matsuda, 2019). In comparison, Switzerland is noticeably decentralized with several cantons and different official languages depending on the region (World Atlas, 2020).

At the same time, Japan and Switzerland share key similarities that make them an interesting comparison. Both countries are experiencing an aging population, as well as the increased burden of cancer and other non-communicable diseases that often comes with such demographic changes; cancer is one of the leading causes of death in both countries, which increases the need to invest in potential solutions such as cancer genomic medicine (Federal Statistical Office, 2021; Nguyen et al., 2022; Federal Statistical Office, 2023). Both countries offer the FoundationOne CDx and FoundationOne Liquid CDx panel tests (Foundation Medicine, 2020; Foundation Medicine, 2022). Finally, both countries have universal healthcare systems in which all residents are required to enroll. This is a distinction from countries like the US where an individual’s health insurance acts as a major factor in access to healthcare and significant inequalities between individuals arise (Mansur et al., 2022a; Mansur et al., 2022b).

The combination of differences and similarities between Japan and Switzerland creates a unique opportunity for comparison. Investigating and comparing patient access to cancer genomic medicine in these two countries not only reveals more nuanced understandings of barriers to patient access and contributes to a broader understanding of the global context, but also identifies shared challenges that are related to the implementation of cancer genomic medicine, regardless of a country’s healthcare or societal context.

In our investigation, we focused specifically on multigene panel testing for cancer patients, including 1) the availability of multigene panel testing in hospitals, 2) the availability of information for patients regarding the possibility of receiving the test, and 3) whether the tests are covered by health insurance. We then discuss the ethical and human rights issues that may arise as a result of certain barriers in these areas, including implications for the future of clinical genomics and health equity in the genomic era.

## 2 Materials and methods

To begin our investigation, we collected primary sources on how multigene panel testing is provided and regulated in Japan and Switzerland. The collection process included four steps. We first began with a desktop-search of official documents published online by the government institutions with jurisdiction over the use of multigene panel testing for cancer in each country, e.g., Japan’s Ministry of Health, Labour, and Welfare (MHLW) and Switzerland’s Federal Office of Public Health (OFSP). We then examined the websites of the major hospitals that provide multigene panel testing for cancer. The hospitals were selected based on the criteria that they act as the central hubs for the practice of cancer genome medicine in their respective regions. In the case of Japan, these hospitals included the thirteen core hospitals for cancer genome medicine designated by the MHLW. In the case of Switzerland, selected hospitals included teaching university hospitals in Zürich, Bern, Geneva, Basel, and Lausanne. We continued our search by exploring national legislation that regulates the practice and reimbursement of multigene panel tests. To find the relevant legislation, we used e-gov.go.jp, a database of laws in Japan, and admin.ch, the national website of Switzerland where all national level legislation is published. Finally, we examined the resources published by the companies that produce the multigene panel tests, e.g., Chugai Pharmaceutical Co., LTD. in Japan (Chugai Pharmaceutical CO LTD, 2023) and Foundation Medicine in Switzerland (Foundation Medicine, 2023).

Secondary sources that analyzed and commented on the use of these tests in Japan and Switzerland were also collected. We began with current scientific and grey literature using search engines such as Google Scholar and PubMed and the following search phrases: (multigene panel testing) AND (cancer OR oncology) AND ([country name]). Experts from each country were also consulted to ensure that no key information was left out.

Based on the collected data, we focused on three areas for comparing the two countries: 1) the availability of multigene panel testing for cancer, 2) the availability and quality of information for patients, and 3) the requirements for insurance reimbursement. Regarding the availability of multigene panel testing, we investigated where testing is offered in the country and considered potential issues in terms of equal access. Regarding the availability and quality of information for patients, we evaluated the ease at which patients would be able to access information regarding the previous two areas, i.e., availability and insurance reimbursement, in terms of the extent to which the information is available and ease of comprehension. After this, we consider its broader implications for patient autonomy. Regarding the requirements for insurance reimbursement, we investigated a) which tests are approved for reimbursement, b) at what stage of treatment they are approved, and c) whether the therapeutic drug identified by the test would be reimbursed by insurance as well. From this, we discuss the implications for the affordability of multigene panel testing for cancer.

## 3 Results

### 3.1 Japan

#### 3.1.1 Test availability

Japan has been working steadily to increase the implementation and uptake of genomic services for precision cancer medicine since 2016, with the most recent initiative being a law to support the development of cancer genomic medicine in the country (E-Gov, 2023). Institutions that offer multigene panel testing for cancer are designated by Japan’s Ministry of Health Labor and Welfare (MHLW). As of January 2024, there are a total of 260 designated hospitals across the country. This includes 13 designated core hospitals (*chuukaku kyoten byouin*), 32 core hospitals (*kyoten byouin*), and 215 cooperative hospitals (*renkei byouin*) (National Cancer Center Hospital, 2023c). Patient samples can be collected at any of the 260 hospitals, though only the designated core and core hospitals can order the tests directly from the main provider of multigene panel tests in Japan, Chugai Pharmaceutical Co., LTD. In the case that patient samples are collected at the cooperative hospitals, the samples are sent to one of the aforementioned hospitals to be analyzed. Expert panel discussions, which discuss the results of the tests and decide on treatment moving forward, are carried out at the designated core and core hospitals. Clinical trials for drug therapy that the test results may indicate are carried out at the designated core hospitals (Ministry of Health and Labour and Welfare MHLW, 2023a; National Cancer Center Hospital, 2023c).

#### 3.1.2 Information for patients

Information for patients regarding multigene panel testing for cancer is available from major stakeholders that provide testing, mainly from Japan’s National Cancer Center Hospital and its associated institutions and resources (National Cancer Center Hospital, 2021; National Cancer Center Hospital, 2023c; Center for Cancer Genomics and Advanced Therapeutics, 2023). Multigene panel testing is explained within the context of cancer genomic medicine and covers topics such as:

• Types of tests that can diagnose genomic mutations (e.g., monogenic testing, multigene panel testing, whole exome sequencing, and whole genome sequencing)
• Process of using multigene panel testing in the clinical field (e.g., informed consent, collection of tissue sample, carrying out the test, expert panel meetings, and return of results)
• Current data regarding the use of the NCC Oncopanel test (e.g., percentage of patients for which actionable genomic mutations are identified and targeted therapeutic drugs are available, 13.4%)
• The flow of genomic information between key institutions such as the designated core, core, and cooperative hospitals, Japan’s data center for genomic medicine known as the Center for Cancer Genomics and Advanced Therapeutics (C-CAT), the Consortium for the Promotion of Cancer Genome Medicine run by the MHLW, research institutions, private companies, *etc.*
• Where panel testing is available, including how activities are coordinated between the designated core, core, and cooperative hospitals
• Therapeutic drugs based on the results of the multigene panel testing, including clinical trials
• Insurance reimbursement

It is also often explicitly indicated whether the information is targeted towards medical professionals, patients, or the family and friends of patients, where the content varies based on the relevance of the information for the target audience. For example, while the content listed above is targeted at patients and the public, information targeted at medical professionals includes information on medical policy, training, and conferences (National Cancer Center Hospital, 2021). There is also information targeted at the family and friends of patients, which includes ways that the individual can support the patient (National Cancer Center Hospital, 2023b).

#### 3.1.3 Insurance reimbursement

All residents of Japan are required to have health insurance, provided either by an employer or Japan’s national health insurance. National health insurance covers a portion of the costs (generally 70%) For medical consultations, medical procedures and treatments, medicines and therapeutic materials, home care and nursing, and hospitalization, patients pay a 30% co-payment while national health insurance covers the remaining 70% of costs. National health insurance is administered by the municipality in which an individual lives and is funded by annual insurance premiums paid by the individual and subsidies from the government (Ministry of Health and Labour and Welfare MHLW, 2017; Ministry of Health and Labour and Welfare MHLW, 2023b).

Five types of multigene panel tests are currently covered by Japan’s national health insurance. The FoundationOne CDx (F1CDx) test was approved for coverage by national health insurance in Japan in June 2019, along with the NCC OncoPanel test. The FoundationOne Liquid CDx (F1 LiquidCDx) was approved 2 years later in September 2021 (Muto, 2020; Naito et al., 2021). Most recently, the Guardant360 CDx test was approved for reimbursement by national health insurance in July 2023 (Guardant Health Inc, 2023), followed by the GenMine TOP test in August 2023 (Konica Minolta, 2023). Under the national health insurance system, patients are required to pay 10%–30% of the fees, which are charged when the test is ordered and when the results are explained to the patient. Fees are not refunded if the test does not yield successful results. In the case that 70% of the costs are covered, the patient may be expected to pay approximately 168,000 yen (1196 USD). Additional fees related to biopsy procedures, genetic counseling, and the drug identified by the test are not included in these costs (National Cancer Center Hospital, 2023a).

Insurance coverage for testing is limited to the following criteria: 1) there is no standard cancer treatment for the patient’s tumor or the patient is at the end of standard cancer treatment without success, and 2) the patient’s attending physician decides the patient would be suitable for receiving a drug therapy that may be indicated by the results of the panel test, based on the guidelines of the relevant academic society (Naito et al., 2021). Patients who do not meet these criteria (e.g., patients in the early stages of cancer treatment) are not eligible for insurance reimbursement. While patients may still receive testing at some hospitals, patients must cover the full costs of the test and related procedures, amounting to upwards of 560,000 yen (3977 USD).

Furthermore, even in the case that the results of a test indicate a specific drug to treat the patient’s specific form of cancer, access to and coverage of the drug is not guaranteed. The drug may be in the stages of a clinical trial, which would not require the patient to pay the cost but may require them to travel far distances to participate. Other drugs may have passed through the clinical trial process but not be approved for insurance coverage (Naito et al., 2021; National Cancer Center Hospital, 2023a).

On top of the aforementioned panel tests that are covered by insurance, some hospitals also offer other additional panel tests such as the PleSSision Panel and Exome tests and the Todai OncoPanel test (Tokyo University Hospital, 2023). These tests as well as related procedures or therapeutic drugs not covered by national health insurance may be covered by private insurance, though that would require the individual having the resources to enroll in it.

### 3.2 Switzerland

#### 3.2.1 Test availability

Comprehensive tumor diagnostics is now becoming increasingly important in everyday clinical practice (Curioni-Fontecedro and Papet, 2020) and is considered standard care (Stoll et al., 2021). In Switzerland, comprehensive molecular tumor profiling is offered by the Institute of Pathology and Molecular Pathology at the University Hospital of Zürich (Universital Hospital of Zürich, 2023). The F1CDx test for solid tumors has been provided in partnership with Foundation Medicine and Roche since 2018 (Den Wert von Daten beleben, 2021), and the F1 LiquidCDx test has been provided by the University Hospital of Zürich since 2023 (Jonsdottir, 2023; Management-Krankenhaus, 2023; Plüss, 2023). Within this collaboration, tumor samples are first sequenced at a molecular profiling laboratory in Switzerland. This is because genetics laboratories and screening services need authorization from the Federal Office of Public Health (Office fédéral de la santé publique OFSP, 2023a), which requires the tests to be performed within the country under the collaboration between Roche and the University Hospital Zürich (Office fédéral de la santé publique OFSP, 2023b). Sequenced data is then sent to Cambridge for bioinformatic analysis by Foundation Medicine. The reports are reviewed by the University Hospital of Zürich, where discussion by a Molecular Tumor Board is also available to evaluate treatment options (Foundation Medicine, 2022).

Other hospitals in Switzerland either utilize a different panel test from a competing company or have developed a test of their own. For example, the Archer FusionPlex NGS panel test is available at the University Hospital of Basel, University Hospital of Lausanne, and University Hospital of Locarno. The Ilumina Pan-cancer Fusion panel is available at the University Hospital of Geneva (Moch et al., 2021).

#### 3.2.2 Information for patients

Information for patients comes mainly from the website of Switzerland’s Federal Office of Public Health (OFSP) as well as the websites of individual hospitals. The OFSP website has a dedicated page available in the three national languages (Office fédéral de la santé publique OFSP, 2022a), which explains the concept of genetic testing on solid tumors and how genetic testing may help to target a better therapy. At the same time, it does not mention the term multigene panel testing and it does not indicate specifically the names of the tests or where each test is available.

The Hospital of Zürich’s website provides an introductory explanation of the test performed in their hospital, the F1CDx test (Universital Hospital of Zürich, 2023). The information is available in German, the official language of the canton of Zürich. This information notably includes:

• Information about the test itself (e.g., validated next genome sequencing-based analysis; number of the genes analyzed; which genetic changes are detected)
• Order form for the test
• Example report
• References

Additional information is available on the FoundationOne Switzerland website. However, it is indicated that the information on this page is “only intended for healthcare professionals $\left[\ldots \right]$ not intended to provide medical advice and/or treatment recommendations” (Foundation Medicine, 2023).

#### 3.2.3 Insurance reimbursement

All residents of Switzerland are required to have basic health insurance. Basic health insurance covers treatments in cases of illness, maternity, and accident, and the same range of benefits are offered to all insured persons. It is assured through around 50 federally recognized non-profit insurers and individuals are free to choose from the insurers operating in their place of residence (Office fédéral de la santé publique OFSP, 2023b). Insurers can also offer “supplementary”/“complementary” insurance to cover special needs and/or additional services (Office fédéral de la santé publique OFSP, 2022b), e.g., a specific insurance plan for “innovative medicine” (Helsana, 2023).

In principle, all genomic sequencing analyses for therapeutic purposes are eligible for coverage by basic health insurance (Hopitaux Universitaires Geneve, 2022; Swiss Institute of Genomic Medicine, 2023). Individuals must pay an ordinary deductible, plus 10% of the cost of the test that exceeds the deductible to a maximum of CHF 700 for adults and CHF 350 for children and adolescents per year (Federal Office of Public Health, 2023). The insurance company may refuse to reimburse the test based on its effectiveness, appropriateness, and economic efficiency (Swiss Academies Communications, 2019; Mon Génome, 2023), though it is unlikely in the case of cancer medicine where the patient has already been diagnosed and needs treatment (Fels, 2019). Looking at the F1CDx test as a specific example, both the University Hospital of Zürich and Roche and FoundationOne formally state that it is not guaranteed that the costs of the test will be reimbursed. However, no practical issues regarding the reimbursement of the test have yet to be reported.

### 3.3 Key findings

This study identifies three critical aspects of the patient access to multigene panel testing for cancer in Japan and Switzerland: 1) test availability, 2) information for patients, and 3) insurance reimbursement. Each of these aspects, along with their implications for equal access, patient autonomy, and healthcare affordability, will be discussed in the following sections.

Data from the following tables are current as of January 2024. Furthermore, we acknowledge that this study is not a comprehensive quantitative analysis of the clinical landscapes in Japan and Switzerland and may leave out minor panel tests developed privately by an individual hospital.

#### 3.3.1 Test availability

**Table:** 

Country	Hospitals	Insured Tests**
**Japan**	• 13 Designated core hospitals • 32 Core hospitals • 215 Cooperative hospitals	• FoundationOne CDx • NCC OncoPanel • FoundationOne Liquid CDx • Guardant360 CDx • GenMine TOP
**Switzerland**	• University Hospital of Zürich • University Hospital of Basel • University Hospital of Lausanne • University Hospital of Geneva • University Hospital of Locarno	• FoundationOne Cdx (Zürich) • Archer FusionPlex NGS (Basel) • Archer FusionPlex NGS (Lausanne) • llumina pan-cancer fusion panel (Geneva) • Archer FusionFlex NGS panel (Locarno)

** List of insured tests for Switzerland is non-exhaustive.

#### 3.3.2 Information for patients

**Table:** 

Country	Source	Content
**Japan**	National Cancer Center Hospital website	• Genetic testing • Multigene panel testing • NCC OncoPanel data • Data infrastructure • Test availability • Drug therapy • Insurance reimbursement
**Switzerland**	Federal Office of Public Health	• Genetic testing • Tumor profiling
	Teaching hospital websites	• Multigene panel testing • Order form • Example report
	FoundationOne Switzerland website	• FoundationOne tests (medical professional- oriented)

#### 3.3.3 Insurance reimbursement

**Table:** 

Country	Coverage	Eligible Tests	Eligibility Criteria
**Japan**	Patients pay 10-30% co-payment; national health insurance covers remaining 70-90%	• FoundationOne CDx • NCC OncoPanel • FoundationOne Liquid CDx • GuardantCDx • GenMine TOP	• There is no standard cancer treatment for the patient’s tumor or the patient has gone through standard cancer treatment without success • The patient’s attending physician decides the patient would be suitable for receiving a drug therapy that may be indicated by the results of the panel test, based on the guidelines of the relevant academic society
**Switzerland**	Patients pay an ordinary deductible plus 10% of costs exceeding deductible; basic health insurance covers remaining costs	All genomic sequencing tests	• The patient’s attending physician decides that the test is for therapeutic purposes (i.e. cancer care) • The patient’s insurance provider approves reimbursement of the test

## 4 Discussion

Results have revealed a broad range of ethical issues related to patient access to multigene panel testing for cancer. Here, we discuss the pros and cons of the two systems and their implications for equal access, patient autonomy, and healthcare affordability in cancer genomic medicine.

### 4.1 Test availability and equal access

First, there are challenges to accessing multigene panel testing for cancer in both countries. Japan presents location-related challenges. Patients can access the five types of insured panel tests at all 260 designated hospitals for cancer genomic medicine. While referral from local clinics may be possible, the 260 designated hospitals are noticeably concentrated in urban areas, limiting access in outlying rural areas. As a significantly smaller country, geographical access may not be as noticeable of an issue in Switzerland. At the same time, Switzerland presents language-based challenges. Hospitals operate using the official language of the canton in which they are located, e.g., German, French, Italian, or Romansh, potentially limiting the ease at which certain patients may access the tests and related services.

These results align with existing literature, highlighting common challenges faced by individuals in rural areas as well as those with language barriers when accessing medical services (National Academies of Sciences, 2018; Best et al., 2022). This points to implications regarding how inequalities manifest in countries that are generally thought of as having high levels of health equality and “universal” healthcare coverage like Japan and Switzerland. Population health is affected by numerous factors, and having national health coverage does not guarantee health equality. In Switzerland, for example, Tzogiou et al. (2021) highlighted barriers faced by migrants when accessing healthcare, including socioeconomic factors such as occupation and income (Tzogiou et al., 2021). Similarly, although Japan is often cited as a prime example of universal health coverage, previous literature has described health disparities among specific groups, including individuals without a steady income, single mothers, and foreign residents (Shirahase and Raymo, 2014; Fujita et al., 2016; Yasukawa et al., 2019). Even in countries with comparatively high levels of health equality and where disparities may not be immediately apparent, the issue of equal access remains a persistent concern.

### 4.2 Information and patient autonomy

Second, the availability of information for patients varies. In Japan, detailed explanations of the tests and their likelihood of success are accessible to patients and the public online, and the information is tailored to specific audiences. However, the relevance of these tests to the individual patient is not clear in terms of whether they may need panel testing or not, and if so, which kind of panel test is the best suited to their situation. In Switzerland, information available to patients and the public is either overly general (e.g., on the OFSP website) or primarily intended for medical professionals (e.g., on the Foundation Medicine website). There is also no public data on the percentage of patients whose test results indicate a potential drug therapy.

These differences are likely due to the different circumstances of each country, where Japan’s numerous sectors (e.g., healthcare, education, *etc.*) tend to be more centralized, while Switzerland has four national languages, is divided into 26 cantons, and belongs to different regional European organizations with different guidelines. While each country has specific needs and it is not necessary for Switzerland to have a centralized resource like Japan, increased information provision may strengthen patient autonomy in making health-related decisions. Such information may include 1) the nature of the test, i.e., under which circumstances it is necessary and the possible outcomes, 2) the kinds of tests that are available, and 3) the differences between each test, including where each test is available and under which conditions they’re reimbursed. This could supplement guidance from medical professionals, strengthening patient autonomy by empowering patients to make more informed choices that have direct impacts on their health.

Furthermore, it is essential to recognize that focusing only on the amount of available information is not sufficient. While there is a greater amount of available information for patients in Japan than in Switzerland, its relevance to each patient’s specific circumstances can be unclear. Simply providing more information does not guarantee that the patient will be more informed. Relevance and clarity are equally important.

### 4.3 Insurance and healthcare affordability

Third, insurance reimbursement policies also exhibit variations. In principle, multigene panel testing is reimbursed by national health insurance in both countries. However, eligibility criteria in Japan requires patients to be at the end of standard cancer treatment. While it is still possible to receive testing without insurance coverage, there is a possibility that the results of these tests are not reviewed by the same expert panels convened by the designated core or core hospitals. In Switzerland, decisions are often at the discretion of the individual’s physician. This may lack clarity from a policy perspective. However, doing so allows a wider range of patients to access affordable testing under the appropriate situations. While it is not necessary for Japan to replicate this method, finding a way to provide insurance reimbursement to early stage patients may benefit patients in Japan.

In both countries, the reimbursement of the test does not include the reimbursement of therapeutic drugs that may be indicated by the results of the test in either country, or there may be certain cases in which the patients have to pay themselves. These results indicate the need to consider the affordability of not only the test itself but also the entire process from diagnosis to treatment. Additional fees related to biopsy procedures, genetic counselling, or the drug identified by the test for further treatment should not be overlooked. Indirect costs should also be considered, particularly those that may affect certain individuals more than others, e.g., travel costs for those in rural or remote areas and income lost during the time required to receive testing for those without steady income. Private insurance may cover these costs, although it is not guaranteed and requires the patient to have the resources to enrol in private insurance in the first place. Furthermore, the increasing trend of privatization of health insurance in Europe and Japan is said to reflect strains on the healthcare system caused by aging populations, an increasingly common trend in numerous countries (Toebes, 2006; Health and Global Policy Institute, 2017).

While it may be ideal to provide reimbursement for services like multigene panel testing, especially in cases of serious health issues like cancer, the burden on the healthcare system and the sustainability of providing insurance coverage for emerging genetic technologies like multigene panel testing must be examined as well. Considering this and the fact that only approximately 10% of patients who undergo the test receive a relevant drug therapy as a result (Sunami et al., 2019; Takeda et al., 2021), the actual affordability of not only the test but the entire process that is required becomes questionable on both individual and systemic levels.

### 4.4 Looking ahead

Despite the existence of universal healthcare in Japan and Switzerland, examining the current state of patient access to cancer genomic medicine in these countries has revealed challenges related to equal access, patient autonomy, and healthcare affordability. This suggests a need to examine a wider range of clinical landscapes to reveal more nuanced understandings of the barriers that individuals in diverse settings may face. On a global level, such settings may include other countries outside the US, UK, and Europe where the current state of cancer genomic medicine is understudied. On a local level, such settings may include communities within a country that experience multiple factors of inequality at the same time (e.g., minority groups and individuals with low socioeconomic status).

Equal access, patient autonomy, and healthcare affordability have not only direct impacts on individual health but also broader implications for health equality and human rights. As such, insights from this study are not limited to Japan and Switzerland and may serve as a reference for other countries also at the early stages of implementing cancer genomic medicine. Furthermore, these insights contribute to the broader discussion on health equality and human rights, which is particularly important in the field of genomics and other rapidly developing biomedical technologies, where careful ethical considerations are necessary to avoid further exacerbating existing inequalities.

### 4.5 Limitations

This study covered only two examples of how patient access to cancer genomic medicine can be affected by various factors, and future research should continue examining a more diverse range of clinical landscapes. Furthermore, this study struggled to capture an unequal amount of information between the two countries since the amount of publicly available information in Switzerland was significantly limited. Lastly, the comparison between the two countries limited our ability to investigate the more specific details of in each country, potentially oversimplifying regional diversities. Future research may benefit from focusing on individual countries and delving deeper into the nuances and intersections of various factors to equal access.

## Data Availability

The original contributions presented in the study are included in the article/Supplementary Material, further inquiries can be directed to the corresponding author.
